# Evolutionary Formation and Distribution of Puumala Virus Genome Variants, Russia

**DOI:** 10.3201/eid2907.221731

**Published:** 2023-07

**Authors:** Ekaterina Blinova, Andrei Deviatkin, Marat Makenov, Yulia Popova, Tamara Dzagurova

**Affiliations:** Central Research Institute of Epidemiology, Moscow, Russia (E. Blinova, M. Makenov);; Russian Academy of Sciences, Moscow (E. Blinova, Y. Popova, T. Dzagurova);; Moscow Institute of Physics and Technology, Dolgoprudny, Russia (A. Deviatkin);; Sechenov First Moscow State Medical University, Moscow (A. Deviatkin)

**Keywords:** Puumala virus, viruses, zoonoses, hemorrhagic fever with renal syndrome, genetic lineage, Clethrionomys glareolus, bank voles, Russia

## Abstract

We analyzed Puumala virus (PUUV) sequences collected from bank voles from different regions of Russia. Phylogenetic analysis revealed PUUV reassortments in areas with the highest hemorrhagic fever with renal syndrome incidence, indicating reassortment might contribute to pathogenic properties of PUUV. Continued surveillance is needed to assess PUUV pathogenicity in Russia.

In Eurasia, hantaviruses cause hemorrhagic fever with renal syndrome (HFRS), which is the most common zoonosis in Russia (*1*). During 2000–2017, a total of 131,590 HFRS cases were reported in Russia (*2*); most (≈98%) HFRS cases are caused by Puumala virus (PUUV) (*2*).

PUUV virions are enveloped particles containing 3 negative-sense single-strand RNA segments (*3*). Those segments vary in length; the large (L) segment is ≈6,550 nt long, the medium (M) segment is ≈3,650 nt, and the small (S) segment is ≈1,828 nt (*4*).

PUUV comprises 8 lineages: Central European, Alpe-Adrian, Danish, South-Scandinavian, North-Scandinavian, Finnish (FIN), Russian (RUS), and Latvian (LAT) (*4*–*6*). Modern PUUV diversity arose from multiple migrations of the viral host, the bank vole (*Clethrionomys glareolus*), during the postglacial period (*7*,*8*); that species also comprises several lineages: Ural, Eastern, Spanish, Italian, Balkan, Western, Carpathian, Basque, and Calabrian (*9*). The LAT, FIN, and RUS PUUV lineages likely had a common ancestor and originated from Eastern refugia (*6*). Those sublineages are carried by *C. glareolus* from Western, Eastern, and Carpathian lineages (*10*,*11*).

The RUS and FIN PUUV lineages are known to exist in Russia (*4*,*8*). Although PUUV-related HFRS has been reported from 54 regions of Russia (*2*), complete protein-coding sequences of the S segment in GenBank are only available from 8 regions: Kursk, Saratov, Samara, Tatarstan, Udmurtia, Bashkortostan, Omsk, and Karelia (Figure 1). Those sequences are mainly from viruses collected in the most epidemiologically active HFRS PUUV hotspots in the Volga region (*2*). PUUV genome variants in 46 other regions of Russia are still unknown. We investigated the distribution of different PUUV genome variants in Russia by obtaining PUUV sequences from bank voles captured in different regions.

**Figure 1:**
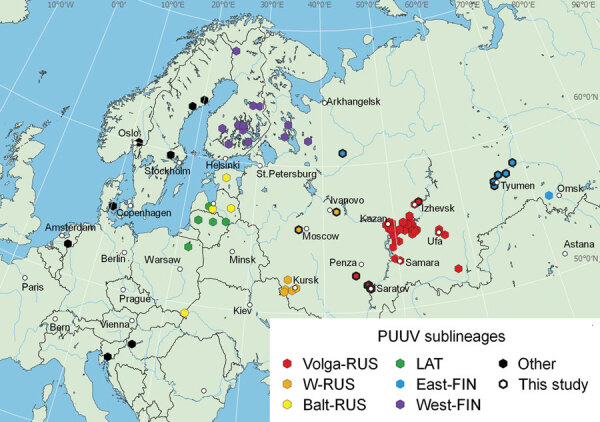
Locations of virus isolation in a study of the evolutionary formation and distribution of Puumala virus genome variants, Russia. The map includes all genome variants belonging to LAT, FIN (West-FIN and East-FIN sublineages), and RUS (W-RUS, Volga-RUS, and Balt-RUS) lineages that had complete coding sequences of the small (S) genome segment available in GenBank as of September 16, 2022. The map was created by using QGIS software (QGIS Development Team, http://qgis.osgeo.org). Color marking for sequences in the map correspond to those in phylogenetic trees (Figure 2). Black frames indicate isolation sites of novel sequences revealed in this study. Because other lineages were not the focus of this study, only a few Puumala virus sequences belonging to other lineages were included. An additional interactive map was generated in R (The R Foundation for Statistical Computing, https://www.r-project.org) by using the leaflet, html widgets, and webshot libraries and is available at https://rpubs.com/andreideviatkin/PUUV_RUS-FIN-LAT_lineages. Balt-RUS, sublineage from the Baltic coast region; East-FIN, sublineage from Siberia and northern Russia; FIN, Finnish lineage; LAT, Latvian lineage; RUS, Russian lineage; Volga-RUS, sublineage from the Volga River Valley; W-RUS, sublineage from western Russia; West-FIN, sublineage from Finland and Russian Karelia.

## The Study

We obtained lung tissue samples from bank voles collected by the territorial Сenters of Hygiene and Epidemiology in the Arkhangelsk, Ivanovo, Moscow, Penza, Saratov, Tyumen, Ulyanovsk, and Udmurtia regions (Figure 1). We stored samples, analyzed hantavirus antibodies by immunofluorescence assay (Table 1), extracted RNA, determined samples with high viral RNA content by real-time reverse transcription PCR (Table 1), and sequenced them as described in previous research (*12*). 

**Table 1:** Virus test results for *Clethrionomys glareolus* bank voles captured and tested in study of evolutionary formation and distribution of Puumala virus genome variants, Russia*

Region	No. trapped	No. IFA-positive	No. PCR-positive
Arkhangelsk	43	10	8
Ivanovo	2	2	2
Moscow	61	12	10
Penza	54	28	27
Saratov	44	14	14
Tyumen	110	10	9
Ulyanovsk	98	7	7
Udmurtia	32	9	9

*IFA, immunofluorescence assay.

To determine bank vole sublineages, we obtained sequences of the *cytochrome b* gene by using a previously described protocol (*13*). In all, we obtained PUUV genomic sequences and *cytochrome b* sequences from 17 bank voles (Appendix Table 1). All voles belonged to the Eastern lineage of *C. glareolus* (Appendix Figure 1).

We mapped locations of the newly obtained PUUV sequences and all representative LAT, FIN, and RUS lineage sequences available in GenBank that had complete S segment coding sequences (Figure 1). We also included representative sequences of other PUUV lineages on the map.

**Figure 2:**
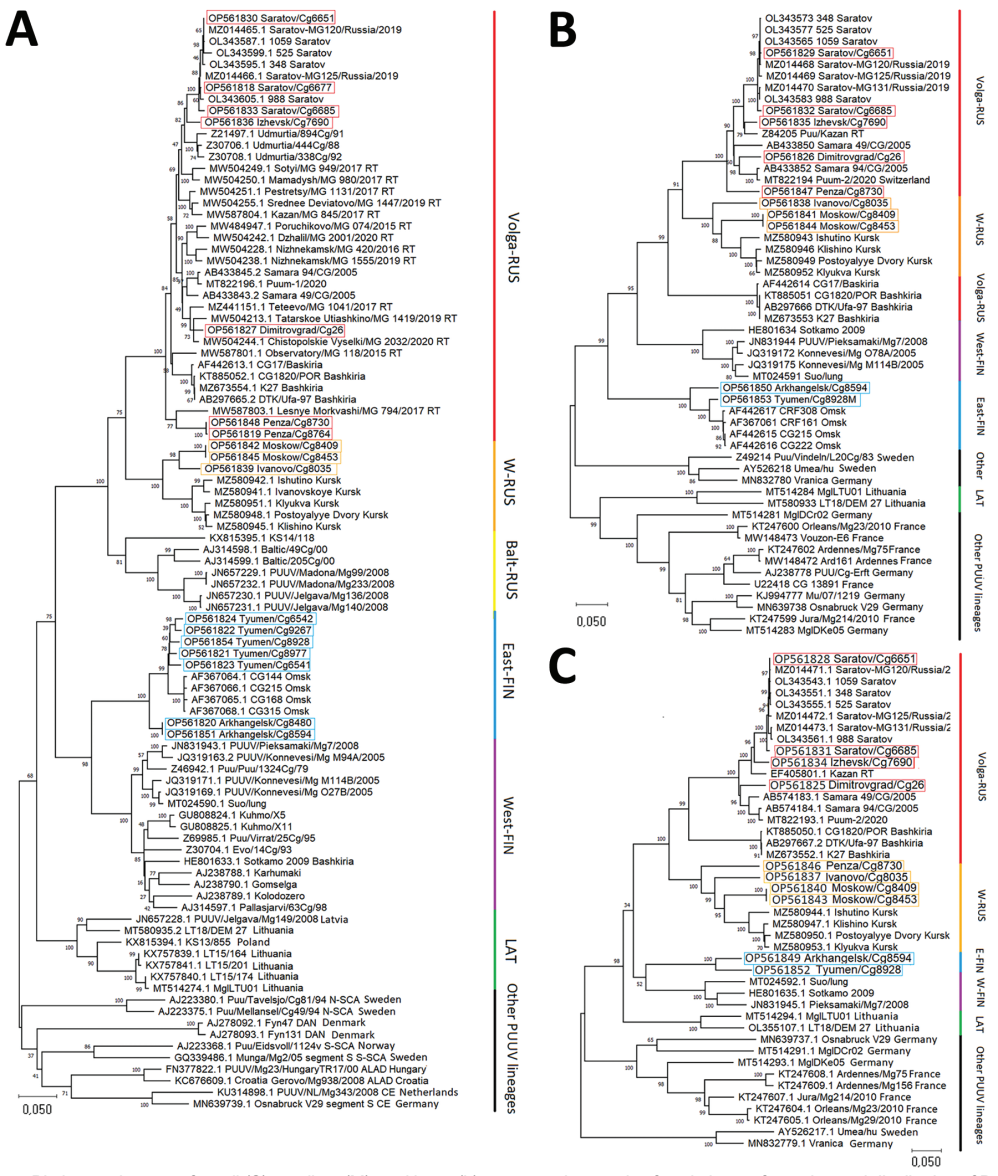
Phylogenetic trees of small (S), medium (M), and large (L) segments in a study of evolutionary formation and distribution of Puumala virus genome variants, Russia. A) S segment based on complete open reading frame (ORF) of 1,302 nt; B) M segment based on partial ORF 2,923 nt (525–3,447 nt of ORF of GenBank accession no. OL343565); C) L segment based on partial ORF 6,405 nt (5–6,409 nt of ORF of GenBank accession no. OL343543). The RUS lineage is divided into 3 large, color-coded subclades: Volga-RUS (red), W-RUS (orange), Balt-RUS (yellow). The FIN lineage is divided into 2 large, color-coded subclades: East-FIN (blue) and West-FIN (purple). Green indicates LAT lineage; black indicates other lineages. Boxes indicated sequences obtained in this study. GenBank accession numbers are provided for all sequences. All alignments and phylogenetic relationships of the sequences were conducted by the MUSCLE algorithm (https://www.ebi.ac.uk/Tools/msa/muscle) and maximum-likelihood method with the general time-reversible model and 1,000 bootstrap by using MEGA version X (https://www.megasoftware.net). The full S segment tree with complete dataset of all available representatives of LAT, FIN, and RUS lineages are available from https://github.com/AndreiDeviatkin/repo/blob/main/S_PUUV.png. Balt-RUS, sublineage from the Baltic coast region; East-FIN, sublineage from Siberia and northern Russia; FIN, Finnish lineage; LAT, Latvian lineage; RUS, Russian lineage; Volga-RUS, sublineage from the Volga River Valley; W-RUS, sublineage from western Russia; West-FIN, sublineage from Finland and Russian Karelia.

For phylogenetic analysis, we added sequences from this study to GenBank representatives of the LAT, FIN, and RUS lineages. We included representatives of the other PUUV lineages as outgroup (*6*) (Figure 2). According to S-segment phylogeny, we propose dividing the RUS lineage into 3 phylogenetically based sublineages: W-RUS (from western Russia) (*12*), Balt-RUS (from the Baltic coast region), and Volga-RUS (from the Volga River Valley). The percentages of identical nucleotides and amino acids within and between the sublineages did not enable us to establish clear criteria for the delimitation of the sublineages according to the quantitative differences (Table 2).

**Table 2:** Percentage identity between sublineages in study of evolutionary formation and distribution of Puumala virus genome variants, Russia*

Sublineage, no. sequences	Volga-RUS, n = 37	W-RUS, n = 8	Balt-RUS, n = 7	East-FIN, n = 11	West-FIN, n = 15	LAT, n = 7
Volga-RUS	97.47–100, 92.93–100	96.77–98.85	95.39–98.16	95.39–97.47	94.01–97.7	95.16–97.24
W-RUS	86.57–90.17	98.62–100, 91.17–100	97.24–98.39	96.77–97.7	94.93–97.93	96.77–97.93
Balt-RUS	85.88–88.86	85.57–88.88	97.93–100, 87.92–100	96.31–97.7	94.70–97.93	96.31–97.93
East-FIN	84.57–86.86	85.17–87.42	85.34–87.39	98.85–100, 94.24–100	96.08–99.08	97.00–97.93
West-FIN	84.27–86.87	85.03–86.77	83.86–86.73	87.02–89.55	96.54–100, 91.01–100	95.39–97.93
LAT	84.38–87.19	85.19–87.27	84.49–86.94	85.93–87.57	84.77–88.23	99.31–100, 89.77–100

*Amino acid and nucleotide identity are shown according to small (S) segment coding sequences. Gray shading indicates nucleocapsid (N) protein calculated by blastp (https://blast.ncbi.nlm.nih.gov), and S complete coding by blastn. Upper triangular matrix indicates identity values based on the complete N protein calculated using the blastp algorithm; lower triangular matrix indicates values based on the S complete coding sequence calculated using the blastn algorithm. Balt-RUS, sublineage from the Baltic coast region; East-FIN, sublineage from Siberia and Russian North; LAT, Latvian lineage; Volga-RUS, sublineage from the Volga River Valley; W-RUS, sublineage from western Russia; West-FIN, sublineage from Finland and Russian Karelia.

The topology of our S-segment tree does not match those from previous studies (*12*). The sequences we obtained provide additional information for better resolution of PUUV. L and S segments of PUUV are comparably conservative (Appendix Figure 2), and their phylogenetic trees mostly repeat each other. The M-segment tree was the most distinct (Figure 2, panel B), a finding that corresponds with previous data (*5*). That finding confirms that the M segment is the most variable and most often involved in reassortment events (*14*).

In the M-segment tree, the W-RUS sublineage stood within the Volga-RUS sublineage, but the branch from Bashkiria formed an outgroup (Figure 2, panel B). Almost the entire Volga-RUS sublineage, except for the Bashkir branch, appears to be a product of the exchange of the М segment between ancestors of the W-RUS and the Bashkir branch. The S and L segments are related to the Bashkir branch, and the M segment was obtained from the ancestors of the W-RUS sublineage. Those findings confirm and extend previous results (*12*). Thus, strains from the most epidemiologically active areas have arisen through reassortment. However, whether reassortment has altered the pathogenic properties of those strains remains unclear.

The genome variant from Penza belongs to the group that obtained the M segment by reassortment, as we have described (Figure 2, panel B). On the S-segment tree, Penza/Cg8730 is located on the Volga-RUS sublineage (Figure 2, panel A). However, on the L-segment tree, Penza/Cg8730 belongs to the W-RUS sublineage (Figure 2, panel C). Thus, Penza/Cg8730 likely emerged from a 2-stage reassortment. Although L segment exchange is not a unique event, it occurs much less frequently than exchange among other segments (*14*). Geographically, the Penza region lies west of locations where viruses belonging to the Volga-RUS sublineage were isolated but far from representatives of the W-RUS sublineage. Thus, Penza could be a meeting point of those 2 lineages.

On the S-segment tree, the FIN branch was represented by 2 sublineages: West-FIN from Finland and Karelia and East-FIN from western Siberia (Figure 2). However, on the M-segment tree, West-FIN and East-FIN did not form a common branch (Figure 2, panel B). The current hypothesis generally accepts that the FIN branch split into 2 parts when the territories were recolonized by bank voles after the glacier melted (*8*).

The sequences we obtained from the Arkhangelsk region clustered with sequences of viruses collected in Omsk and Tyumen regions, despite their geographic distance (Figure 1; Appendix Figure 3). According to the phylogenetic trees, the Tyumen and Omsk clades divided after the Arkhangelsk clade split from their common ancestor (Figure 2). Such branching suggests 2 possible paths for spread of the East-FIN subclade: from northwest Russia to the southeast, across the Ural Mountains to western Siberia; or spreading from the Pre-Ural region in 2 directions, to western Siberia and to the Arkhangelsk region. The second possibility supposes a second vole migration wave to the north, after PUUV split off from the East-FIN and West-FIN groups during postglacial period (*8*). PUUV sequences from the area between Arkhangelsk and western Siberia might shed more light on spread of the FIN lineage.

## Conclusions

The newly identified viruses from the Tyumen and Arkhangelsk regions of Russia and viruses from Omsk form a common East-FIN branch. This finding raises additional questions about the dispersal routes of the FIN lineage.

Here we considered the RUS lineage as 3 separate sublineages: Volga-RUS, W-RUS, and Balt-RUS. The PUUV genome variants from Tatarstan, Udmurtia, Samara, and Saratov probably emerged through reassortment because they contained S and L segments related to the Bashkir branch and an M segment derived from ancestors of the W-RUS sublineage. The Penza/Cg8730 genome variant might have arisen from a 2-stage reassortment. Continued surveillance is needed to assess PUUV pathogenicity in Russia, but we found PUUV reassortments in areas with the highest HRFS incidence, indicating reassortment might contribute to pathogenic properties of PUUV. 

Appendix Additional information on the evolutionary formation and distribution of Puumala virus genome variants, Russia.

## References

[1] Dzagurova TK, Siniugina AA, Ishmukhametov AA, Egorova MS, Kurashova SS, Balovneva MV, et al. Pre-clinical studies of inactivated polyvalent HFRS vaccine. Front Cell Infect Microbiol. 2020;10:545372. 10.3389/fcimb.2020.545372 33251155 PMC7673229

[2] Tkachenko EA, Ishmukhametov AA, Dzagurova TK, Bernshtein AD, Morozov VG, Siniugina AA, et al. Hemorrhagic fever with renal syndrome, Russia. Emerg Infect Dis. 2019;25:2325–8. 10.3201/eid2512.181649 31742540 PMC6874259

[3] Petazzi RA, Koikkarah AA, Tischler ND, Chiantia S. Detection of envelope glycoprotein assembly from old-world hantaviruses in the golgi apparatus of living cells. J Virol. 2021;95:e01238–20. 10.1128/JVI.01238-20 33239451 PMC7851546

[4] Kabwe E, Davidyuk Y, Shamsutdinov A, Garanina E, Martynova E, Kitaeva K, et al. Orthohantaviruses, emerging zoonotic pathogens. Pathogens. 2020;9:775. 10.3390/pathogens9090775 32971887 PMC7558059

[5] Razzauti M, Plyusnina A, Niemimaa J, Henttonen H, Plyusnin A. Co-circulation of two Puumala hantavirus lineages in Latvia: a Russian lineage described previously and a novel Latvian lineage. J Med Virol. 2012;84:314–8. 10.1002/jmv.22263 22170553

[6] Castel G, Chevenet F, Razzauti M, Murri S, Marianneau P, Cosson JF, et al. Phylogeography of Puumala orthohantavirus in Europe. Viruses. 2019;11:679. 10.3390/v11080679 31344894 PMC6723369

[7] Saxenhofer M, Weber de Melo V, Ulrich RG, Heckel G. Revised time scales of RNA virus evolution based on spatial information. Proc R Soc B Biol Sci. 2017;284(1860):20170857. 10.1098/rspb.2017.0857 PMC5563803 28794221

[8] Sironen T, Vaheri A, Plyusnin A. Molecular evolution of Puumala hantavirus. J Virol. 2001;75:11803–10. 10.1128/JVI.75.23.11803-11810.2001 11689661 PMC114766

[9] Drewes S, Ali HS, Saxenhofer M, Rosenfeld UM, Binder F, Cuypers F, et al. Host-associated absence of human Puumala virus infections in northern and eastern Germany. Emerg Infect Dis. 2017;23:83–6. 10.3201/eid2301.160224 27983499 PMC5176216

[10] Jeske K, Schulz J, Tekemen D, Balčiauskas L, Balčiauskienė L, Hiltbrunner M, et al. Cocirculation of *Leptospira* spp. and multiple orthohantaviruses in rodents, Lithuania, Northern Europe. Transbound Emerg Dis. 2022;69:e3196–201. 10.1111/tbed.14470 35119222

[11] Rosenfeld UM, Drewes S, Ali HS, Sadowska ET, Mikowska M, Heckel G, et al. A highly divergent Puumala virus lineage in southern Poland. Arch Virol. 2017;162:1177–85. 10.1007/s00705-016-3200-5 28093611

[12] Blinova E, Deviatkin A, Kurashova S, Balovneva M, Volgina I, Valdokhina A, et al. A fatal case of haemorrhagic fever with renal syndrome in Kursk Region, Russia, caused by a novel Puumala virus clade. Infect Genet Evol. 2022;102:105295. 10.1016/j.meegid.2022.105295 35526822

[13] Schlegel M, Ali HS, Stieger N, Groschup MH, Wolf R, Ulrich RG. Molecular identification of small mammal species using novel cytochrome B gene-derived degenerated primers. Biochem Genet. 2012;50:440–7. 10.1007/s10528-011-9487-8 22193288

[14] Klempa B. Reassortment events in the evolution of hantaviruses. Virus Genes. 2018;54:638–46. 10.1007/s11262-018-1590-z 30047031 PMC6153690

[15] Kumar S, Stecher G, Li M, Knyaz C, Tamura K. MEGA X: molecular evolutionary genetics analysis across computing platforms. Mol Biol Evol. 2018;35:1547–9. 10.1093/molbev/msy096 29722887 PMC5967553

