# Supplementary material for: Evolutionary Formation and Distribution of Puumala Virus Genome Variants, Russia
Source: Emerg Infect Dis. 2023 Jul;29(7):1420–4. doi: 10.3201/eid2907.221731 (PMC10310372; doi:10.3201/eid2907.221731)
Supplement: Appendix — Additional information on the evolutionary formation and distribution of Puumala virus genome variants, Russia. [file 22-1731-Techapp-s1.pdf]

Article DOI: https://doi.org/10.3201/eid2907.221731

EID cannot ensure accessibility for supplementary materials supplied by authors. Readers who have difficulty accessing supplementary content should contact the authors for assistance.

Evolutionary Formation and Distribution of Puumala Virus Genome Variants, Russia

Appendix

Appendix Table. Genome sequences obtained in a study of evolutionary formation and distribution of Puumala virus genome variants, Russia*

	Clethrionomys glareolus†		Puumala virus		
Isolation region	No.	GenBank accession no.	Segment	Length, nt	GenBank accession no.
Ulyanovsk, Dimitrovgrad	26	OQ885024	L	6,405	OP561825
			M	3,629	OP561826
			S	1,780	OP561827
Saratov	6651	OQ885025	L	6,405	OP561828
			M	3,629	OP561829
			S	1,773	OP561830
	6685	OQ885027	L	6,405	OP561831
			M	3,629	OP561832
			S	1,773	OP561833
	6677	OQ885026	S	1,773	OP561818
Udmurtia, Izhevsk	7690	OQ885028	L	6,405	OP561834
			M	3,654	OP561835
			S	1,774	OP561836
Ivanovo	8035	OQ885029	L	6,405	OP561837
			M	3,628	OP561838
			S	1,806	OP561839
Moscow	8409	OQ885030	L	6,405	OP561840
			M	3,629	OP561841
			S	1,780	OP561842
	8453	OQ885031	L	6,405	OP561843
			M	3,629	OP561844
			S	1,780	OP561845
Penza	8730	OQ885034	L	6,405	OP561846
			M	3,628	OP561847
			S	1,777	OP561848
	8764	OQ885035	S	1,777	OP561819
Arkhangelsk	8594	OQ885033	L	6,402	OP561849
			M	3,079	OP561850
			S	1,790	OP561851
	8480	OQ885032	S	1,790	OP561820
Tyumen	8928	OQ885036	L	6,405	OP561852
			M	3,107	OP561853
			S	1,780	OP561854
	6542	OQ885040	S	1,781	OP561824
	6541	OQ885039	S	1,779	OP561823
	9267	OQ885038	S	1,780	OP561822
	8977	OQ885037	S	1,781	OP561821

*L, large; M, medium; nt, nucleotide; S, small.
†*Cytochrome b* sequences were used to identify *C. glareolus* as the host species.

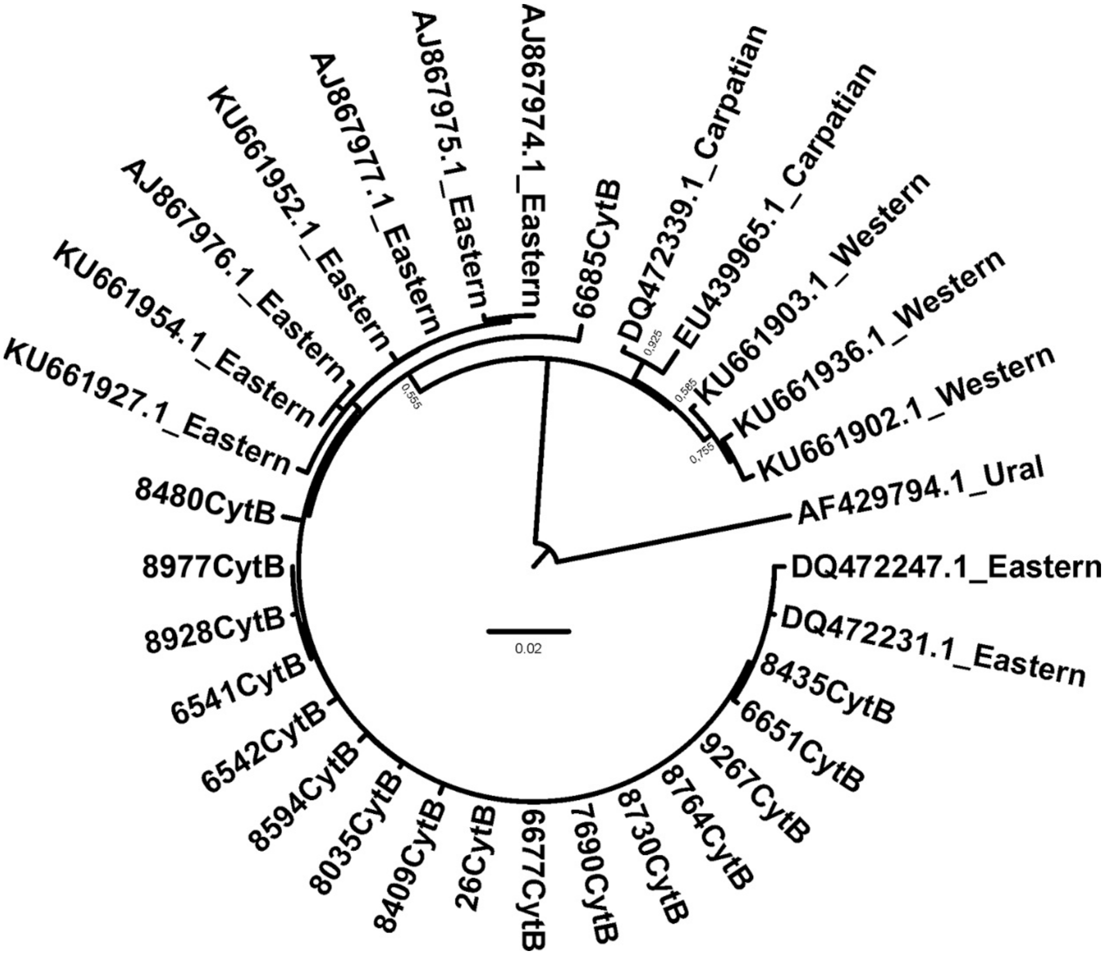

Appendix Figure 1. Phylogenetic analysis of fragment of *cytochrome b* based on alignment of 907 nt from 17 bank voles (*Clethrionomys glareolus*) used in a study of Puumala virus genome variants, Russia. Obtained sequences were supplemented by representatives of Eastern, Western, Carpstian, and Ural lineages from previous research (*1*). Alignment and phylogenetic relationships of the sequences were conducted by the MUSCLE algorithm and Maximum-likelihood method with Tamura-Nei model (G) with 1,000 bootstraps by using MEGA version X (https://www.megasoftware.net).

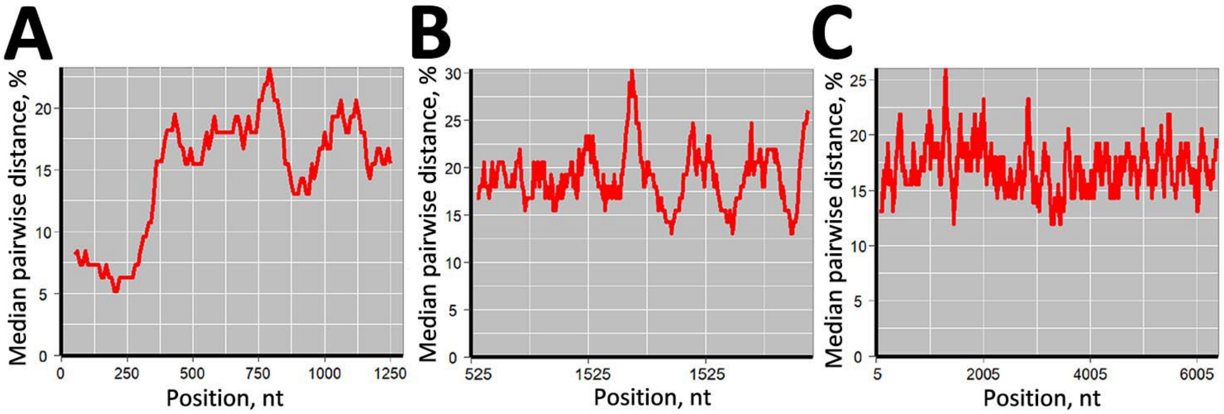

Appendix Figure 2. Conservation plot of Puumala virus segments. A) S segment full coding sequence 1,302 nt long. B) M segment partial coding sequence 525–3,447 nt of ORF. C) L segment partial coding sequence 5–6,409 nt of ORF. The coding sequence of N protein (S segment) contains overlapping ORF of nonstuctural protein of S segment (NSs) is located from 41 to 311 nt, which corresponds to a conservative region. Mean pairwise genetic distance was calculated using R (R Foundation for Statistical Computing, https://www.r-project.org), with window size 100 nt and step 10 nt. L, large; M, medium; ORF, open reading frame; S, small.

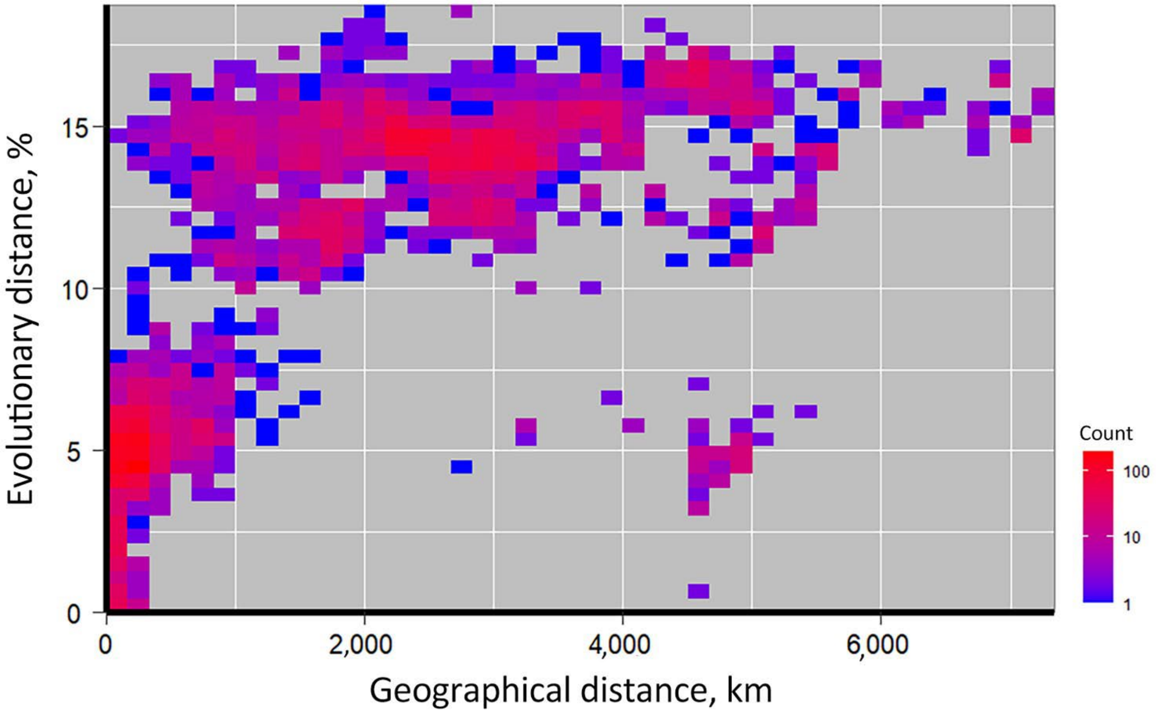

Appendix Figure 3. Pairwise distribution of evolutionary and geographic distance of Puumala virus (PUUV) genome variants. The distribution of phylogenetic and geographic distance for each pair of PUUV sequences are shown. The main part of the points corresponds to a high degree of divergence (pairs

from different lineages) and another range characterized by high relation and geographic proximity. The main interest is a small point cloud, which stands out from the main graph and corresponds to pairs with a distant geographic distance and close relation. That cloud includes pairs containing sequences from the Arkhangelsk region and representatives of the FIN branch (East-FIN subclade). Pairwise distribution of evolutionary and geographic distance was calculated using R (R Foundation for Statistical Computing, https://www.r-project.org).

Reference

1. Drewes S, Ali HS, Saxenhofer M, Rosenfeld UM, Binder F, Cuypers F, et al. Host-associated absence of human Puumala virus infections in northern and eastern Germany. Emerg Infect Dis. 2017;23:83–6. PubMed https://doi.org/10.3201/eid2301.160224
